# Supplementary material for: The Impact of Drug Interactions in Patients with Community-Acquired Pneumonia on Hospital Length of Stay
Source: Geriatrics (Basel). 2022 Jan 4;7(1):11. doi: 10.3390/geriatrics7010011 (PMC8788264; doi:10.3390/geriatrics7010011)
Supplement: Supplementary file 1 [file geriatrics-07-00011-s001.zip › geriatrics-1464609-supplementary.pdf]

Supplementary Materials

Table S1. Results of the regression analysis of hospital LOS and different factors for patients aged <65 years.

Variable	RR	95% CI of RR	*p* VALUE
Intercept	4.87		
PCCL (ref: 0)			<0.001
1	0.98	0.69–1.38	
2	1.12	0.84–1.50	
3	1.38	1.10–1.72	
4	1.89	1.39–2.58	
No. of drug interaction	1.01	0.8–1.05	0.376
Intensity of drug interactions (ref: none/mild)			0.003
moderate, severe, and/or contraindicated	1.40	1.13–1.75	

PCCL = German Patient Clinical Complexity Level, CI = confidence interval. SE = standard error, ref = reference.

Table S2. Results of the regression analysis of hospital LOS and different factors for patients aged between 65 years and 79 years.

Variable	RR	95% CI of RR	*p* Value
Intercept	5.52		
PCCL (ref: 0)			<0.001
1	1.09	0.84–1.41	
2	1.48	1.21–1.82	
3	1.25	1.03–1.51	
4	1.17	1.76–2.69	
No. of drug interaction	1.02	0.99–1.04	0.239
Intensity of drug interactions (ref: none/mild)			0.075
moderate, severe, and/or contraindicated	1.18	0.98–1.42	

PCCL = German Patient Clinical Complexity Level, CI = confidence interval, SE = standard error, ref = reference.
